# Sensory Processing of Motor Inaccuracy Depends on Previously Performed Movement and on Subsequent Motor Corrections: A Study of the Saccadic System

**DOI:** 10.1371/journal.pone.0017329

**Published:** 2011-02-23

**Authors:** Muriel Panouillères, Christian Urquizar, Roméo Salemme, Denis Pélisson

**Affiliations:** INSERM U1028, CNRS UMR5292, Lyon Neuroscience Research Center, IMPACT (Integrative, Multisensory, Perception, Action and Cognition) Team and University Lyon 1, Lyon, France; Istituto di Neuroscienze, Italy

## Abstract

When goal-directed movements are inaccurate, two responses are generated by the brain: a fast motor correction toward the target and an adaptive motor recalibration developing progressively across subsequent trials. For the saccadic system, there is a clear dissociation between the fast motor correction (corrective saccade production) and the adaptive motor recalibration (primary saccade modification). Error signals used to trigger corrective saccades and to induce adaptation are based on post-saccadic visual feedback. The goal of this study was to determine if similar or different error signals are involved in saccadic adaptation and in corrective saccade generation. Saccadic accuracy was experimentally altered by systematically displacing the visual target during motor execution. Post-saccadic error signals were studied by manipulating visual information in two ways. First, the duration of the displaced target after primary saccade termination was set at 15, 50, 100 or 800 ms in different adaptation sessions. Second, in some sessions, the displaced target was followed by a visual mask that interfered with visual processing. Because they rely on different mechanisms, the adaptation of reactive saccades and the adaptation of voluntary saccades were both evaluated. We found that saccadic adaptation and corrective saccade production were both affected by the manipulations of post-saccadic visual information, but in different ways. This first finding suggests that different types of error signal processing are involved in the induction of these two motor corrections. Interestingly, voluntary saccades required a longer duration of post-saccadic target presentation to reach the same amount of adaptation as reactive saccades. Finally, the visual mask interfered with the production of corrective saccades only during the voluntary saccades adaptation task. These last observations suggest that post-saccadic perception depends on the previously performed action and that the differences between saccade categories of motor correction and adaptation occur at an early level of visual processing.

## Introduction

The brain monitors and maintains its performance using error signals. For example, when an individual repeatedly produces inaccurate movements, subsequent movements can be progressively modified so that they land closer to their goal. Such sensori-motor adaptation is elicited when a discrepancy between movement endpoint and target position repeats itself over trials. In principle, signals providing movement error information are provided either by sensory feedback about the trajectory and/or endpoint of the on-going movement (sensory error signal) or by feedforward motor commands of fast corrective responses (motor error signal - see for review [1]). These two possibilities are not mutually exclusive and sensory and motor error signals may simultaneously contribute to the adaptive re-calibration of a given motor response. This is because motor adaptation in general is a complex function comprising both unconscious/implicit and strategic/explicit mechanisms [2]. Saccadic adaptation is a well-established model of implicit motor adaptation which provides insight into motor plasticity mechanisms independently of strategic responses (see for reviews [3]–[5]). An additional advantage of the saccadic system over other motor systems is the clear separation between the motor amendments induced by error signals, namely the adaptive motor recalibration and the fast motor correction, which can be respectively addressed by measures of the primary saccade and of corrective saccades. In the classical target double-step protocol used to study saccadic adaptation [6], a saccadic error is artificially generated by systematically displacing the visual target during saccade execution. Beside the adaptive change of primary saccade amplitude which develops when such trials are repeated, this target displacement triggers secondary saccades which correct for the induced error. It has been found that saccadic adaptation can take place even if no such corrective saccades are produced during the adaptation phase [7]–[9]. This indicates that error signals driving saccadic adaptation are not primarily of motor origin. Thus, although they may not be purely sensory either (see [7]), error signals necessary to induce adaptation strongly depend on post-saccadic visual feedback. How such post-saccadic visual information contributes to error signals for adaptation is however virtually unknown and will be the main topic of the present paper.

Recent studies in monkeys [10] and in humans [7], [11], showed that optimal saccadic adaptation requires that the target displacement eliciting visual error occurs shortly after (within ∼100 ms) primary saccade completion. Shafer et al. [10] also studied the relationship between the post-saccadic duration of the displaced target and the amount of adaptation. They found that the visual target must be maintained for at least 80 ms after saccade termination to produce a strong adaptation. To our knowledge, no such study has been conducted in human, although saccadic adaptation differs in several aspects between human and monkey: indeed, it develops faster and is more specific relative to saccade categories in human [11]–[13] than in monkey [14], [15].

Two main categories of saccades can be defined: reactive saccades are externally triggered by the brisk appearance of a new target in the environment, whereas voluntary saccades are internally triggered by the subject and are aimed to explore objects already present in the environment. It is well-known that the production of these two types of saccades involves separate neural substrates [16]–[21]. In addition, recent evidence in human indicate that different mechanisms are involved in the adaptation of reactive and voluntary saccades [12], [22]–[26] but not in monkey [14]. However, it has never been studied whether adaptation of different categories of saccades results from common or specific visual error signals.

As mentioned above, the systematic target perturbation of the adaptation paradigm triggers corrective saccades, at least during the initial phase of adaptation time-course. However, except for the demonstration that they are not essential for the adaptation of reactive saccades [7]–[9], corrective saccades have not been investigated so far in adaptation studies. Thus, whether the control of corrective saccades relies on the same error processing mechanisms as primary saccades adaptation is unknown.

The objective of the present study was to test in human subjects, both for reactive and voluntary saccades, the characteristics of error signals leading to saccadic adaptation and to the generation of secondary corrective saccades. To this aim, the double-step target paradigm [6] was slightly modified to induce an adaptive gain decrease of both saccade categories [12]. In this paradigm, the subject makes a saccade toward a visual target and, when the primary saccade is detected, the target is displaced toward the initial position of the eyes. Repeating this intra-saccadic target step over about 100 successive trials leads to a progressive decrease of saccade gain. This gain change results from implicit adaptive processes because subjects are usually unaware of the intra-saccadic target displacement (saccadic suppression phenomenon) and because the saccade gain change persists after completion of the double-step exposure phase. In the present study, the processing of error signals was experimentally altered by manipulating the post-saccadic visual feedback in two ways. First, the post-saccadic duration of the displaced visual target was varied in different adaptation sessions. Second, in some adaptation sessions, the displaced target was followed by a visual mask that interfered with visual target processing. We predicted that these manipulations of post-saccadic visual feedback 1) would affect adaptation and corrective saccades, similarly in the reactive and in the voluntary saccade adaptation tasks but possibly, 2) would interfere differently with adaptation mechanisms and with corrective saccades production.

## Results

Reactive and voluntary saccades were adapted in separate sessions and each session was divided in three phases: pre-adaptation, adaptation and post-adaptation. We will first report on the characteristics of primary saccades during the pre-adaptation phase. In this phase (composed of 1 block of 24 saccades), the target was replaced at saccade onset by a mask or a blank screen, in the mask and no-mask condition, respectively. The effects of saccade categories and of visual masking will be determined.

### Primary saccades in pre-adaptation phase: effects of saccade type and of visual masking

The latency of saccades in pre-adaptation was submitted to a two-way ANOVA testing the “visual masking” (mask vs no-mask) and “saccade type” (reactive vs voluntary) factors. No effect of the “visual masking” factor was found whereas the effect of “saccade type” factor was strongly significant (F[1,116] = 86.7; p<0.001). As expected, the latencies of reactive saccades were significantly shorter than the latencies of voluntary saccades (199.7±3.7ms and 416.3±21.2 ms, respectively; post-hoc Fisher's LSD test, p<0.001). Saccades gain was identical for the two types of saccades and did not depend on the presence of the visual mask (mean value: 0.90±0.01; F[1,116]<0.0004, p>0.29). In contrast, for saccadic duration, the “saccade type” factor and the interaction between “visual masking” and “saccade type” factors were significant (F[1,116] = 5.39; p<0.05 and F[1,116] = 8.8, p<0.001; Table 1). This was due to a significantly longer duration of voluntary saccades in the mask condition compared to all other combinations of saccade type and condition (post-hoc Fisher's LSD test, p<0.01). Finally, for saccadic peak velocity, we found a significant interaction of the “visual masking” and “saccade type” factors (F[1,116] = 5.5, p<0.05). This effect was related to reactive saccades in the mask condition being significantly faster than in all other combinations (post-hoc Fisher's LSD test, p<0.05), but not faster than voluntary saccades in the no-mask condition.

**Table 1:** Baseline of saccade duration and peak velocity (pre-adaptation phase).

		Mask	No-mask
Duration (ms)	Reactive	36.5±0.7	37.9±1.2
	Voluntary	**41.5±0.9** **	37.3±0.9
Velocity (°/sec)	Reactive	**305.9±5.8** *	280.6±7.9
	Voluntary	287.4±7.6	**298.5±7.0**

Values are mean ± SEM. Mean duration and peak velocity were calculated for all subjects of the mask and no-mask condition (with the saccade directions pooled together). For these two parameters, a significant interaction of “visual masking” and “saccade type” factors was detected (see text). The values in bold font with an asterisk (*) are statistically different from those in regular font (post-hoc Fisher's LSD test, * p<0.05, ** p<0.01).

In summary, the latency of voluntary saccades was longer than that of reactive saccades, as previously reported (see e.g. [12]). Whereas saccadic gain did not differ between saccade types and between conditions (mask vs no-mask), the visual mask appears to moderately increase the duration of voluntary saccades and the peak velocity of reactive saccades.

**Figure 1:**
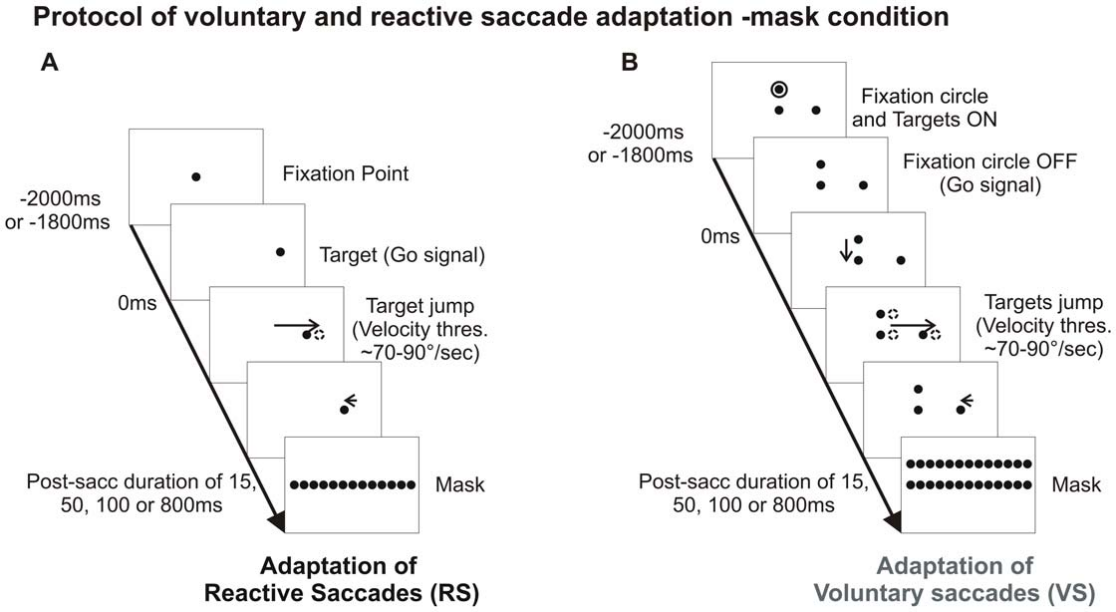
Protocol of reactive and voluntary saccade adaptation in the mask condition (only rightward trials represented). Vertical and long horizontal arrows indicate primary saccades and short horizontal arrows indicate corrective saccades. When a horizontal primary saccade is detected (threshold of 70–90°/sec) the target or the display jumped, respectively in the reactive and voluntary saccade adaptation. Fifteen, 50, 100 or 800 ms after saccade offset, the visual display is replaced by a mask in the mask condition. In the no-mask condition, 15 or 50 ms after saccade termination, a blank screen replaced the visual display.

The pre-adaptation phase was immediately followed by the adaptation and the post-adaptation phases. The adaptation phase was composed of 2 blocks of trials with an intra-saccadic target step representing 25% of initial target eccentricity and of 2 blocks with a 40% target step (see Figures 1A and 1B for the adaptation protocol of reactive and of voluntary saccades, respectively). Error signals were tested by setting the post-saccadic duration of stepped target (hereafter called “target duration”) at 15, 50, 100 and 800 ms in separate sessions and, in some sessions, by replacing the stepped target by a visual mask. The time-course of the adaptation and the occurrence of corrective saccade during the adaptation phase were assessed for the different target durations and visual masking conditions. The post-adaptation phase was identical to the pre-adaptation phase (no intra-saccadic target step), allowing us to determine the after-effect of the adaptation as the gain change in post-adaptation relative to pre-adaptation.

### Primary saccades in adaptation and post-adaptation phases: effects of saccade type, of target post-saccadic duration and of visual masking

One of the goals of this study was to interfere with error signals processing by varying the post-saccadic duration of jumped target and by using a visual mask; and to test the effect of these interferences on the adaptation of reactive and voluntary saccades. To quantify adaptation for each target duration and each masking condition (mask vs no-mask), the gain change relative to pre-adaptation was averaged across the 5 subjects for each block of trials of the adaptation and post-adaptation phases.

#### Mask condition

**Figure 2:**
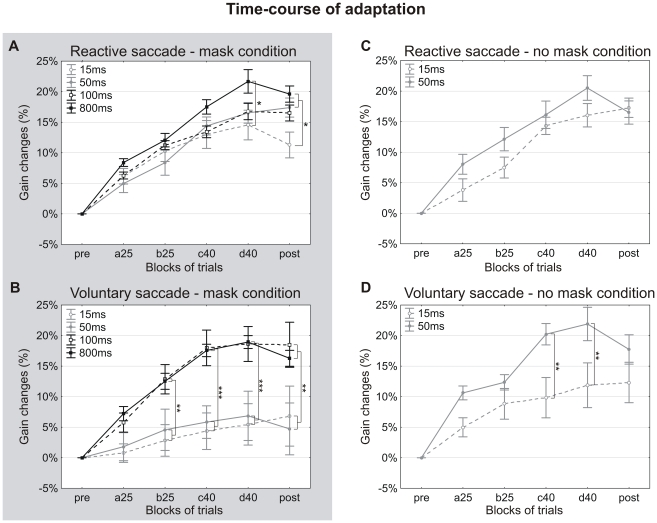
Time-course of the adaptation of reactive and voluntary saccades. Mean gain change is represented as a function of the blocks of trials and superimposed for the different post-saccadic durations of jumped target in the mask condition (grey background – A, B) and no-mask condition (white background – C, D), for reactive (A, C) and voluntary (B, D) saccades adaptation. Mean gain change was calculated across the 5 subjects of each experimental session. Gray lines indicate the gain changes for the shortest target durations (dashed lines: 15 ms – solid lines: 50 ms) and black lines indicate the gain changes for the longest target durations (dashed lines: 100 ms – solid lines: 800 ms). The blocks of trials are: pre-adaptation (pre), adaptation blocks with an intra-saccadic step of 25% of initial target eccentricity (a25, b25) or of 40% (c40, d40) and post-adaptation (post). Error bars are SEMs. Significant differences of gain changes between the target durations are indicated by * (p<0.05), ** (p<0.01) and *** (p<0.001).

The time-courses of the gain changes are presented for reactive and voluntary saccades in Figures 2A and 2B, respectively. The saccadic adaptation is shown superimposed for the different target durations. The depicted increase of gain changes across successive blocks of trials revealed a progressive decrease of gain during the adaptation phase. For reactive saccade adaptation, the gain changes seemed to be lower for the shortest target duration than for other target durations, but only in the last adaptation block and in the post-adaptation block. Conversely, the adaptation of voluntary saccades is strongly impaired for the two shortest target durations (15 and 50 ms), throughout all the adaptation phase and the post-adaptation block. Thus, the adaptation seems to depend on the target duration, but also on the category of saccades. To quantify this, the mean gain changes were submitted to a three-way ANOVA testing the “block” (pre, …post), “saccade type” (reactive vs voluntary), and “target duration” factors (15 ms vs 50 ms vs 100 ms vs 800 ms). Significant effects of all three factors were found (F[5,432] = 63.8, p<0.001; F[1,432] = 24.1, p<0.001 and F[3,432] = 28, p<0.001 respectively). A strong interaction between the “target duration” and the “saccade type” factors was also found (F[3,432] = 10.2, p<0.001), and the interaction between “target duration” and “block” factors just reached significance (F[15,432] = 1.7, p = 0.05). The effect of the “block” factor resulted from a progressive decrease of saccade gain in the adaptation and post-adaptation blocks for all target durations and for the two types of saccades (Figures 2A and 2B). The other results of the ANOVA indicated that these adaptive gain changes depended both on saccade type and on target duration. For the shortest durations of target (15 and 50 ms), the gain of reactive saccades showed a larger decrease than the gain of voluntary saccades for the last two blocks of adaptation (c40 and d40) and the post-adaptation block (“after-effect”) (post-hoc Fisher's LSD test, p<0.05). In contrast, no significant difference between the gain changes of reactive and voluntary saccades was highlighted for the long target durations (100 ms and 800 ms). For reactive saccades, the gain changes differed between the longest (800 ms) and the shortest (15 ms) target durations only for the last block of adaptation and the post-adaptation block (post-hoc Fisher's LSD test, p<0.05). Contrary to this, for voluntary saccades, gain changes differed between the two shortest durations (15 ms and 50 ms) and the two longest durations (100 ms and 800 ms) of jumped target for all adaptation blocks but a25 and for the post-adaptation block (post-hoc Fisher's LSD test, p<0.01 and p<0.001).

To summarize, for reactive saccades, the target duration of 15 ms led to a smaller adaptation, than the target duration of 50 ms and the latter led to optimal adaptation (i.e. adaptation did not improve with further increases of target duration). In comparison, for voluntary saccades, the smallest target duration that produced a normal adaptation was 100 ms. In view of these results, we asked if this difference between reactive and voluntary saccades depended entirely on the presence of the visual mask. To answer this question, adaptation of reactive and voluntary saccades was further tested in a no-mask condition, for the two target durations (15 and 50 ms) where the largest difference of adaptation was found between the two saccade types.

#### No-mask condition

The time-courses of the mean gain changes are presented in Figure 2C (reactive saccades) and Figure 2D (voluntary saccades). Data for target durations of 15 ms and 50 ms are shown superimposed. In the no-mask condition, the gain of reactive saccades similarly decreased for both target durations. In contrast for voluntary saccades, stronger gain changes are induced with a target duration of 50 ms than of 15 ms. A four-way ANOVA with the “block”, “saccade type”, “target duration” (15 ms vs 50 ms) and “visual masking” (mask vs no-mask) factors disclosed a significant effect of all 4 factors on gain changes (F[1,420]>14.5, p<0.001). Significant interactions between “saccade type” and “visual masking” factors and between “target duration” and “visual masking” factors were also found (respectively, F[1,420] = 5.6, p<0.05 and F[1,420] = 20.8, p<0.001). As previously noted, the effect of the “block” factor originates from the progressive reduction of gain relative to pre-adaptation in all adaptation and post-adaptation blocks. The other results of the ANOVA indicate a strong dependency of adaptation on saccade type, target duration and visual masking. For reactive saccades, gain changes observed for both target durations (15 and 50 ms) were quite similar between the mask and the no-mask conditions. However, with the 15 ms target duration, the gain change in post-adaptation relative to pre-adaptation (“after-effect”) tended to be smaller in the mask (11.3±1.6%) than in the no-mask condition (17.3±1.0% - post-hoc Fisher's LSD test, p = 0.06). For voluntary saccades, the adaptation after-effect differed significantly between the two conditions for the 15 ms target duration (post-hoc Fisher's LSD test, p<0.05). Moreover, when the target remained visible for 50 ms, gain changes for all adaptation and post-adaptation blocks were much larger in the no-mask condition than in the mask condition (post-hoc Fisher's LSD test, p<0.05). For this target duration, adaptation after-effect was only of 4.8±4.2% in the mask condition, whereas it reached 17.7±2.4% in the no-mask condition. Thus, in the no-mask condition, it appears that a strong adaptation of reactive saccades is reached when the jumped target remains visible for only 15 ms after primary saccade, whereas a strong adaptation of voluntary saccades requires a target duration of 50 ms.

**Figure 3:**
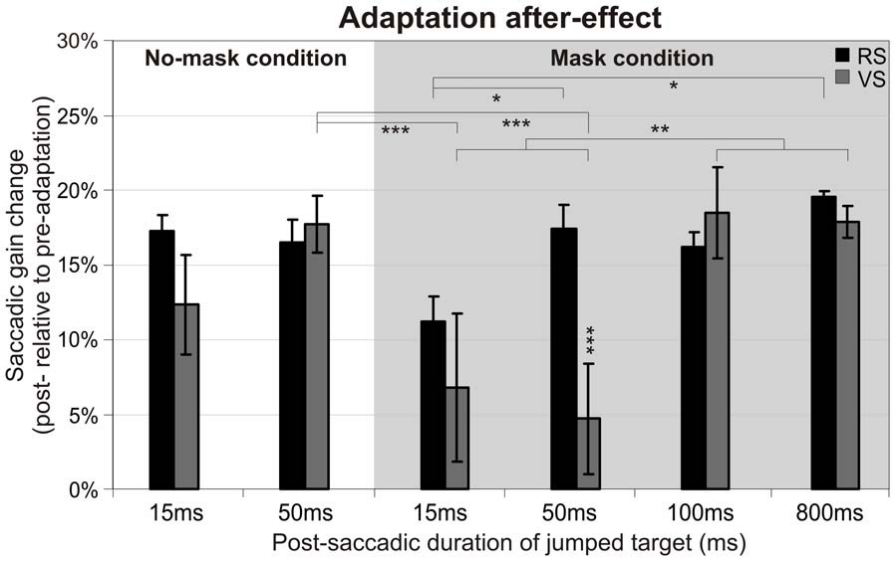
Adaptation after-effect for the different target durations and mask conditions. Mean gain changes for reactive saccades (black bars) and voluntary saccades (grey bars) calculated between the pre-adaptation and post-adaptation blocks. These gain changes are plotted as a function of target durations in the mask condition (grey background) and in the no-mask condition (white background). Error bars are SEMs. Significant differences of gain changes between the saccade categories, target durations and masking conditions are indicated by * (p<0.05), ** (p<0.01) and *** (p<0.001).

Figure 3 is a summary of the adaptation after-effects obtained for the two saccade types, the two masking conditions and the different target durations. As previously mentioned, optimal saccadic adaptation in the mask condition is achieved with a target duration of 50 ms for reactive saccades and of 100 ms for voluntary saccades. In the no-mask condition, the corresponding minimal values necessary for optimal adaptation are 15 ms and 50 ms. To sum up, the temporal integration of post-saccadic visual information for eliciting saccadic adaptation depends both on the type of saccades and on the presence of a visual mask.

### Secondary saccades in adaptation phase: effects of saccades type, of target post-saccadic duration and of visual masking

**Figure 4:**
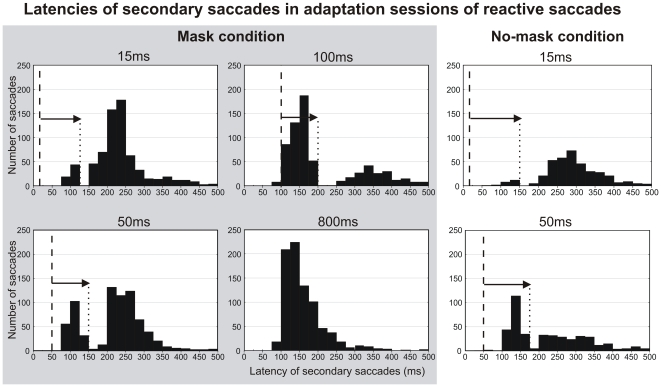
Latencies of secondary saccades in adaptation sessions of reactive saccades. Latency distribution of secondary saccades (first saccades following primary saccades, whether corrective or not) measured in the adaptation sessions of reactive saccades. Different target durations (15, 50, 100 and 800 ms) are shown in the different plots for the mask condition (grey background) and the no-mask condition (white background). Note a dip in distributions (arrow and dotted lines) around 100 ms (mask condition) or 125–135 ms (no-mask condition) after the disappearance of the stepped target (vertical dashed line).

Secondary saccades are defined as the first saccade following each primary horizontal saccade. Figure 4 represents latency distributions of secondary saccades generated during the adaptation phase of reactive saccades. Overall, fewer secondary saccades were produced in the no-mask condition than in the mask condition (also true for voluntary saccades, data not shown). Moreover, when the target duration increased, the latencies of secondary saccades tended to be smaller. This is best shown in the mask condition where a wider range of target durations was tested, but this effect can also be noted in the no-mask condition. These latency differences are associated with differences in the shape of the distribution. Whereas for the 800 ms target duration, the distribution has the classical uni-modal and asymmetrical shape skewed toward long latencies, for all other tested durations a bi-modal distribution emerges as a consequence of missing responses within a restricted time window (corresponding to one or two bins of 25 ms). This “dip” in the latency distribution started around 100 ms after disappearance of the jumped target in the mask condition and around 125–135 ms in the no-mask condition. Note also that in general, few secondary saccades occurred with latencies shorter than 100 ms.

**Figure 5:**
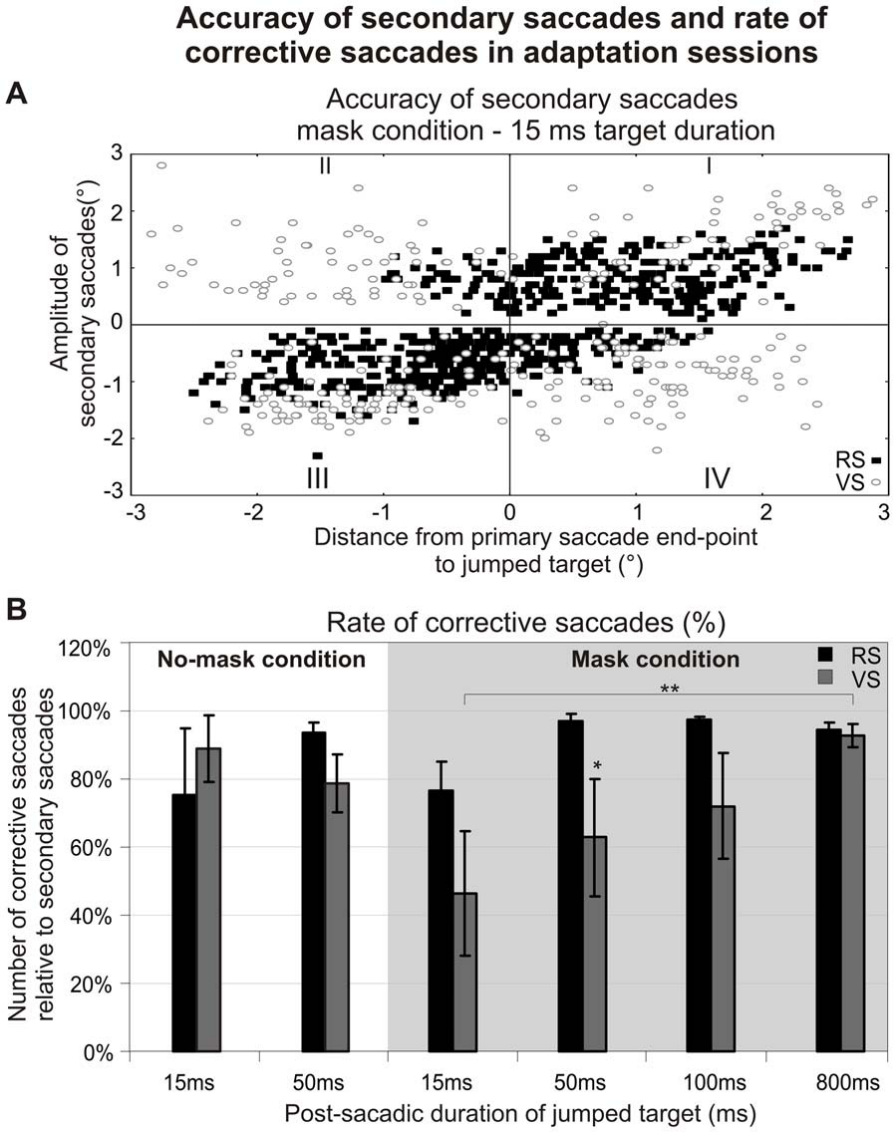
Accuracy of secondary saccades and rate of corrective saccades in adaptation sessions. (A) Amplitude of secondary saccades represented as a function of distance from primary saccade endpoint to jumped target. In this example, the secondary saccades were measured in the adaptation sessions of reactive (▪) and of voluntary (o) saccades in the mask condition, for the target duration of 15 ms. Secondary corrective saccades are located in quadrants I and III, corresponding to secondary saccades with positive (negative) amplitude when the jumped target was located in the right (left) visual field. (B) Rate of corrective saccades relative to the total number of secondary saccades measured during the adaptation sessions of reactive (black bars) and voluntary (grey bars) saccades. The mean rate of corrective saccades was calculated across the 5 subjects of each experimental session and is represented as a function of the target durations (15, 50, 100 and 800 ms) for the mask (grey background) and the no-mask (white background) conditions. Error bars are SEMs. Significant differences of gain changes between the saccade categories and target durations are indicated by * (p<0.05), ** (p<0.01).

We will now focus on the production of secondary saccades directed to the jumped target (corrective saccades) as an oculomotor measure of the visual processing of the stepped target. In Figure 5A, the amplitude of secondary saccades is plotted as a function of the distance from primary saccade end-point to jumped target, with data of reactive and voluntary saccades adaptation sessions shown superimposed (15 ms - mask condition). To avoid anticipatory responses not elicited by the stepped target, only secondary saccades with a latency comprised between 100 ms and 500 ms were plotted. In this plot, corrective saccades are located in quadrants I and III, corresponding to secondary saccades with positive (negative) amplitude when the jumped target was located in the right (left) visual field. Note in Figure 5A, that more corrective saccades were produced during the adaptation of reactive saccades than of voluntary ones. Figure 5B presents the mean values across 5 subjects of the rate of occurrence of such corrective saccades relative to the total number of secondary saccades. In the mask condition, a two-way ANOVA established an effect of the “saccade type” and “target duration” factors on the amount of corrective saccades (respectively, F[1], [32] = 8.66, p<0.01 and F[3], [32] = 3.06, p<0.05). The “saccade type” effect corresponded to a higher rate of occurrence of corrective saccades during the adaptation of reactive saccades than of voluntary saccades, for the 15, 50 and 100 ms jumped target durations (only significant for the 50 ms duration; post-hoc Fisher's LSD test, p<0.05). The “target duration” effect resulted from a general increase of the rate of occurrence of corrective saccades with the target duration. For voluntary saccades, this increase is continuous and progressive between 15 and 800 ms whereas for reactive saccades, it occurred abruptly between 15 and 50 ms. A three-way ANOVA was aimed to seek the effect of the “visual masking”, “saccade type” and “target duration” (15 ms vs 50 ms only) factors on the amount of corrective saccades. This ANOVA did not show any significant effect but only a trend for the “saccade type” factor (F[1], [32] = 3.42, p = 0.07). Thus, for the short target durations (15 and 50 ms), neither the adapted saccade type, nor the visual masking significantly influenced the amount of corrective saccades.

To conclude, the latency distribution of secondary saccades disclosed a dip that followed the time of target disappearance by 100 ms to 135 ms. Regarding the effect of target duration and visual mask on corrective saccade generation, only the shortest target duration (15 ms) in the mask condition and for voluntary saccade adaptation was associated with a reduced rate of corrective saccade.

## Discussion

This study was designed to better understand how the brain monitors and improves motor performance. Movement accuracy was experimentally altered by systematically displacing the visual target during motor execution. Two responses to this perturbation are generated by the central nervous system: a fast motor correction toward the re-located target and a progressive adaptation of motor programming across subsequent trials. Studying the saccadic system provided the advantage of a clear separation between the measures of the fast motor correction (corrective saccade production) and of the adaptive motor recalibration (primary saccade modification), allowing to test whether similar or different error signals are involved in these two processes. The visual component of such error signals was manipulated by varying the duration of the jumped target and by applying a visual mask.

**Figure 6:**
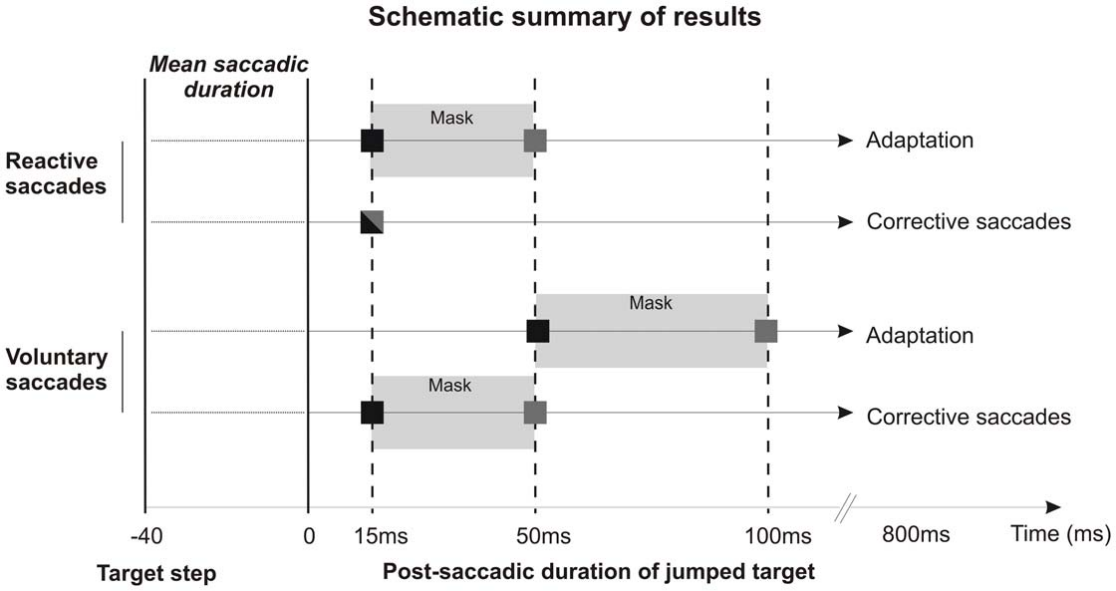
Schematic representation of the results. This schema represents the differences in error signal processing between saccade categories and between adaptation and corrective saccade generation. Each square indicates the minimal target duration leading to optimal adaptation or to optimal generation of corrective saccades, for both saccade categories. The shading of the square represents the masking condition in which this target duration is required: grey square for the mask condition and black square for the no-mask condition (bicolour square when the same target duration is required in both masking conditions). The grey rectangles “Mask” symbolise the fact that when the mask is presented, a longer duration of stepped target is necessary to induce optimal adaptation and corrective saccade generation.

The main finding of this study was that these manipulations affected saccadic adaptation and corrective saccades generation in different ways and that these effects differed between reactive and voluntary saccades. First, we found that applying a visual mask just after target presentation led to increase the minimum target duration necessary to get optimal adaptation. Second, under both the mask and no-mask conditions, the adaptation of reactive saccades unexpectedly required a shorter target duration than the adaptation of voluntary saccades. Third, the mask interfered with the generation of secondary corrective saccades only for voluntary saccades. Finally, although saccadic adaptation and corrective saccades production both depended on visual masking and saccade type, corrective saccades production was quantitatively less affected by these factors than saccadic adaptation (see Figure 6).

### Latency of secondary saccades and saccadic inhibition

Even if not directly related to the main topic of this study, the effect of visual masking and of variations of target duration on the latency of secondary saccades is an original finding that we will discuss first. We found that the latency of secondary saccades decreased when the post-saccadic target duration increased, which is similar to the observation made by Shafer et al. (2000) for corrective saccades in their monkey study of reactive saccades adaptation. Thus, both in human and in monkey, a longer post-saccadic duration of target is associated with a shorter latency of secondary saccades. A similar relationship between saccade latency and target duration has been previously described for primary saccades [27]. This could be accounted for by an improvement of visual processing with longer target durations, such as an increase of the signal-to-noise ratio. Additionally in our study, this relationship between secondary saccade latencies and target duration could be related to the bi-modal latency distribution observed for short target durations (from 15 to 100 ms). Indeed, a “dip” lasting some 25 to 50 ms occurred about 100 ms after target disappearance in the mask condition and about 125–135 ms in the no-mask condition. Because of this transient inhibition, some secondary saccades could have been post-poned, further increasing the latency distribution asymmetry (see in Figure 4 the slight increase of the “tail” of the 15-50-100 ms latency distributions relative to the 800 ms distribution). To our knowledge, this inhibition phenomenon has never been reported for secondary saccades but only for primary saccades [28], [29]. A dip in the saccade latency distribution was observed 100 ms after the presentation of a task-irrelevant visual flash, leading the authors to conclude: “Saccadic inhibition may serve to give the brain time to process the arrival of abrupt changes in visual input by delaying the execution of saccades” [28]. The current study further suggests that this inhibition is a more general phenomenon related to visual transients because 1) it also affects secondary saccades which rely on different motor decision mechanisms than primary saccades, and 2) it is observed in both the mask and no-mask conditions, indicating that the sole disappearance of the jumped target is a change in visual input salient enough to trigger inhibition.

### Differences between human and monkey in minimal duration of error signals driving optimal adaptation of reactive saccades

The present study disclosed that the minimal duration of the post-saccadic error signal necessary for an optimal adaptation of reactive saccades is 15 ms in human (no-mask condition). Shafer et al. [10] found a corresponding duration of 80 ms in the monkey. In our study, 196 trials were used to adapt saccades (98 in each direction) with two target jumps (25 and 40% of initial target eccentricity). In their study, Shafer et al. used ∼1700 trials (∼850 trials in each direction) with an intrasaccadic target jump of 30%. In view of the fact that only ∼100 saccades are sufficient for adaptation to asymptote in human [30]–[34] whereas ∼1000 trials are necessary in monkey [15], the amounts of trials used in Shafer et al. 's study and in ours seem optimal to reach a strong adaptation. In Shafer et al. 's study, the target jump occurred at the end of the saccade whereas in the present study, the target jumped at saccade onset. Thus, for any value of post-saccadic duration, the total duration of stepped target was increased in our study by a value corresponding to the saccadic duration (about 40 ms). This extra-time would have favoured adaptation only if the processing of visual information about the displaced target can start during the saccade, despite the saccadic suppression phenomenon. The study of Gaveau et al. [35] demonstrated that a target step occurring at saccade onset can significantly modify the on-going trajectory of large saccades (∼30 deg). Another study, investigating automatic corrections of hand pointing movements [36], showed that presenting a displaced visual target only during the saccadic response period led to significant updating of the hand's trajectory. Thus, these two studies suggest that some visual processing of the jumped target position can be initiated during the saccade. In the present study a similar intra-saccadic visual processing could have contributed to the stronger saccadic adaptation than in the Shafer et al.'s study. Testing this hypothesis would require reducing the post-saccadic target duration to very short values. Unfortunately, we could not examine values shorter than 15 ms because of the unavoidable delay of image presentation related to the refresh rate of the screen (140 Hz, i.e. a new image every 7 ms) and to the delay of the on-line processing of eye-tracker signals. Yet, even if we consider the possibility that visual information is processed during the saccade, then the corrected value of minimum duration of the jumped target leading to optimal adaptation of reactive saccades in our human subjects (∼55 ms) is still shorter than for non-human primates (80 ms). This suggests a more efficient visual processing in human than in monkey for the induction of saccadic adaptation. Thus the current study highlights a new difference between human and monkey for saccadic adaptation, adding to the known differences of time-course and of transfer patterns (see Introduction). A more efficient visual error processing could partly explain the faster time-course of adaptation in human than in monkey.

### Error signals processing leading to the adaptation differs between reactive and voluntary saccades

Several studies of adaptation transfer showed that reactive and voluntary saccades rely on separate adaptation mechanisms. Studies of the transfer of adaptation between these two saccade categories showed that adaptation of one saccade category does not fully transfer to the other saccade category and that the pattern of transfer is usually asymmetrical [11]–[13], [23], [26], [37], [38]. The conclusion that specific adaptation mechanisms are involved for these two saccade categories is further supported by the pattern of adaptation transfers to arm reaching movements and to anti-saccades. Indeed, several studies have revealed that adaptation of reactive saccades does not –or very little– transfer to hand pointing movements [23], [39]–[41]. In contrast, Cotti et al. [23] recently demonstrated that adaptation of voluntary saccades does significantly transfer to arm movements. In addition, testing the transfer to anti-saccades revealed different patterns for the two saccade categories [24], [25], [42]. Whereas adaptation of reactive saccades transferred only to anti-saccades with the same motor vector as the adapted saccade, adaptation of voluntary saccades also transferred to anti-saccades with the same sensory vector as the adapted saccade. These studies provide complementary lines of evidence supporting the hypothesis that the adaptation of reactive saccades involves late stages (motor) of sensory-motor transformation whereas the adaptation of voluntary saccades also involves early stages (sensory). Finally, a recent study of cerebellar patients suggested that different cerebellar territories participate in the adaptation of reactive and of voluntary saccades [22].

All the aforementioned articles highlighted that the adaptation processes for reactive and voluntary saccades are different and may involve separate neural substrates. These differences concern the adaptive recalibration of oculomotor commands induced by persistent saccadic error. However, whether the visual processes which encode this error information also depend on saccade type is completely unknown, and so far there was no reason to expect any specificity. Contrary to this expectation, the present study shows for the first time that the computation of error signals leading to adaptation differs between saccade types. Indeed, irrespective of whether a visual mask was present, voluntary saccades required a longer visual feedback to reach a similar level of adaptation as reactive saccades (Figure 6). Thus quite surprisingly, the post-saccadic visual processing for the adaptation depends on the category (reactive or voluntary) of the primary saccade completed just a few tens of milliseconds earlier. The fact that the generation of corrective saccades also depends on the type of the just-completed primary saccade, as will be discussed in the next paragraph, further suggests that this saccade specificity takes place at an early level of visual processing. Our observations suggest that the perception is tightly linked to the previously performed action. A recent study showed that the effect of saccadic adaptation on localization of flashed or stationary probes also depends on the type of adapted saccade (reactive versus scanning – [38]), consistent with the idea that this saccade specificity takes place at an early level of visual processing. The question that arises in the present study is why do the post-saccadic visual processing leading to adaptation of the two saccade categories differ? One explanation may be related to the fast initiation of reactive saccades. In ecological conditions, reactive saccades might be expected to reach as fast and precisely as possible a new object that suddenly appears in the visual field, because this new object could vanish as abruptly as it appeared (see for example [43]). We propose that this oculomotor urgency could be complemented by a perceptual urgency speeding-up post-saccadic visual processing specifically after completion of reactive saccades. In comparison, this sense of urgency may not exist for voluntary saccades because they are generated at the subject's self-pace between sustained targets.

### Error signals processing leading to the generation of corrective saccades differs between reactive and voluntary saccades

Corrective saccades are automatic movements aimed at reducing the discrepancy which remains after a primary saccade between eye and target positions. Thus, there was no reason a priori to expect a difference of corrective saccades production between reactive and voluntary saccades adaptation tasks. Without mask, the shortest duration of jumped target (15 ms) was sufficient to fully induce corrective saccades in the reactive and voluntary adaptation tasks. The mask interfered with corrective saccades specifically in the voluntary adaptation task, inhibiting their production in the 15 ms condition (Figure 6). As a consequence, a difference of corrective saccade generation between saccade categories appeared in the mask condition. Because we could not test a shorter duration than 15 ms, we cannot exclude the possibility that the mask would have interfered with the production of corrective saccades also for the reactive saccades adaptation. But even in this case, the visual feedback necessary to reach a similar rate of corrective saccades would still be longer in the voluntary saccades task than in the reactive saccades task. Because this difference was only present in the mask condition, it could result from a stronger effect of the mask in the case of voluntary saccades adaptation. Indeed, this mask was more complex (two lines of dots) than the one used for reactive saccades adaptation (one line of dots), and could have more efficiently prevented the visual processing of stepped target position. Additionally and similarly to the explanation proposed above for the difference of adaptation, the difference of corrective saccade generation between the two saccade tasks could also be directly related to the type of primary saccade (reactive vs voluntary) that has just been produced.

### Error signals processing differs between saccadic adaptation and corrective saccades generation

In this study, we can report a few cases in which a given visual input (defined by the target duration and the masking condition) was responsible for a high rate of corrective saccades but for a less than optimal adaptation (Figure 6). For instance, for voluntary saccades in the no-mask condition, a target duration of 15 ms is sufficient to generate corrective saccades but not good enough to induce a strong adaptation. Moreover, visual masking seems to interfere with the adaptation more than with the generation of corrective saccades. For example, in the case of reactive saccades, a longer duration of the stepped target is necessary in the mask condition to induce a strong adaptation than to produce corrective saccades.

Thus, the manipulation of error signals processing affects both saccadic adaptation and corrective saccades production, but to different extents, and with different amounts of interaction with saccade tasks. Our results highlight a dissociation between saccadic adaptation and corrective saccades production and thus confirm that corrective saccades are not necessary to drive adaptation [7]–[9]. This dissociation also suggests that different processing mechanisms of post-saccadic error signals are involved for saccadic adaptation and for corrective saccades generation. This could be related to the involvement of different neural substrates. Another possibility is that these different error processing mechanisms are performed by the same neural network but produce neural signals characterized by different levels of robustness and information content (i.e. error signals may be more reliable for the immediate preparation of a corrective saccade than for the delayed adaptive change of subsequent saccades). Further studies are required to test these possibilities.

This study showed that error signals processes leading to saccadic adaptation and to corrective saccade production are affected by the temporal characteristics of the visual target information and by the masking of this information. Moreover, the error signals processing inducing the adaptation depends on the type of saccade that has just been performed: a longer duration of target is required for voluntary saccades adaptation to reach the same level as reactive saccades adaptation. Finally, a given error information does not affect adaptation and corrective saccade generation in the same way. Thus, different brain mechanisms monitoring error signals are involved for the immediate control of motor performance though motor corrections and for the progressive improvement of movement accuracy through plastic re-calibration of motor programming. Brain mechanisms monitoring error signals also depend on the type of movement initiation. This suggests that the perception is tightly linked to the previously performed action and that the differences between saccade categories of motor correction and adaptation occur at an early level of visual processing.

## Materials and Methods

### Subjects

Thirty-eight volunteers took part to this study (mean age: 27.6±6.9 years, 18 women, 32 fully naïve subjects). All subjects had a normal or corrected to normal vision. Several subjects participated to different sessions but with a gap of at least one week between 2 experimental sessions.

### Ethics Statement

The study conformed with the Code of Ethics of the World Medical Association (Declaration of Helsinki) and all procedures were approved by the INSERM U864 ethics committee. According to French law, the INSERM U864 ethics committee considered that a written consent was not necessary and that a verbal consent was sufficient for the present behavioural and non-invasive study. Before taking part to an experimental session, the experimenter explained the subjects the duration of the session (∼25minutes) and the task they would have to perform (fast and accurate eye movements to track dots). All subjects gave their informed verbal consent to participate to the study.

### Apparatus

The experiment took place in a dark room with the subjects seating 57 cm from a 140 Hz computer screen (size: 30°×40° of visual angle) controlled by a Visual Stimuli Generation system (CRS Cambridge, UK). Head movements were restrained by a chin rest, a forehead rest and cheekbone rests. The subjects were asked to follow visual targets (0.6° black disks on a grey background) shown on the computer screen.

The horizontal and vertical positions of each eye were recorded with an infrared video eye tracker (Eyelink II, SR Research, Canada), with a frequency of 250 Hz and a spatial resolution of 0.05°. The calibration of the eye tracker was performed before each experimental session by asking the subject to look at 9 targets forming a rectangle covering the screen (28° high ×38° wide). In-house software allowed monitoring of eye movement data both for off-line analysis and for on-line change of the visual display synchronised to the primary saccade (detection of this saccade was based on a velocity threshold of 70−90°/sec).

### Behavioral task

Adaptation of reactive saccades and of voluntary saccades was induced using double-step target protocols similar to those described by Alahyane et al [12]. To test the temporal characteristics of the error signal processing which lead to saccadic adaptation and to corrective saccade generation, the post-saccadic duration of the jumped target was varied and set at 15, 50, 100 or 800 ms in different sessions. The interference with visual target processing was further increased by replacing the jumped target by a visual mask (see below for details about this mask condition). For the 15 and 50 ms target durations, strong differences of adaptation were detected between reactive and voluntary saccades in this mask condition (see Results). To investigate if these differences could be explained only by the presence of the mask, we tested for these 15 and 50 ms durations a “no-mask” condition in which the jumped target was simply replaced by a blank image.

In summary, in the reactive saccade experiment, there were 4 experimental sessions for the mask condition (target duration of 15, 50, 100 and 800 ms followed by a mask) and 2 experimental sessions for the no-mask condition (target duration of 15 and 50 ms followed by a blank). The same 6 experimental sessions were performed in the voluntary saccade experiment. Altogether, 12 experimental sessions were thus performed with 5 subjects per experimental session.

### Reactive saccade experiment—Adaptation phase

At the beginning of a trial, the subject looked at a central fixation point (FP) for 1600, 1800 or 2000 ms (Figure 1A). After this time, the FP was turned off and replaced by a target located at +8° or −8° along the horizontal meridian. When the primary saccade onset was detected (eye velocity reaching a 70–90°/sec threshold), the target switched position. This intra-saccadic target step was directed toward the fixation point to induce a decrease of saccade amplitude and corresponded to 25% of the initial target eccentricity for the first 2 blocks of 48 trials (a25 and b25 blocks) and to 40% for the last 2 blocks of 48 trials (c40 and d40 blocks). In the mask condition, 15, 50, 100 or 800 ms after the primary saccade offset was detected (velocity below a 70–90°/sec threshold), the stepped target was replaced by a visual mask for 500 ms. In the no-mask condition, 15 or 50 ms after the detection of primary saccade offset, the jumped target was turned off and replaced by a blank screen for 500 ms. Then in both conditions, a blank screen was displayed and a beep indicated the participants to direct their gaze back to the centre of the screen to get prepared for the next trial.

The visual mask used in the reactive saccades experiment was a line, displayed on the horizontal meridian, composed of dots identical to the targets and separated by 0.1° (Figure 1A). None of the dots from the mask corresponded to the target location before or after the jump.

### Reactive saccade experiment—Pre and post-adaptation

Two identical blocks preceded (pre-adaptation) and followed (post-adaptation) the adaptation phase. They were similar to the adaptation blocks except that, when the primary saccade onset was detected (velocity threshold: 70–90°/sec), the target did not jump but instead was replaced by a mask or a blank screen in the mask and no-mask conditions, respectively. Each pre- and post-adaptation block is composed of 12 rightward trials and 12 leftward trials.

### Voluntary saccade experiment—Adaptation phase

At the beginning of the trial, the subject looked at a FP displayed 4° above the horizontal meridian (Figure 1B). Then 1600 ms later a circle appeared around the FP simultaneously with 2 targets: one target located 4° below the FP (centre of the screen) and the other one located +8° or −8° lateral to this target. Five hundred ms later, the disappearance of the circle signalled the subject to make first a vertical saccade toward the central target and then a second horizontal saccade to look at the lateral target. When the onset of the horizontal –voluntary – saccade was detected (eye velocity reaching a 70–90°/sec threshold), the entire visual display was shifted horizontally toward the screen centre. The intra-saccadic display step presented the same properties as the target step of the reactive saccade experiment. After a given post-saccadic duration (similar to reactive saccade adaptation), the shifted display was replaced by a mask or a blank screen in the mask and no-mask conditions, respectively. To enforce horizontal voluntary saccades as much as possible, the participants were asked to identify the letter located inside the central target, namely a normal ‘E’ or a truncated ‘E’ (2 pixels missing), and to report at the end of the trial the number of truncated ‘E’ (0 or 1).

The visual mask used in the voluntary saccade experiment was composed of 2 lines of dots: one on the horizontal meridian and one 4° above, at the level of the FP (Figure 1B). The dots were separated by 0.1° and none of them was located at the same position as the lateral target before or after the jump.

### Voluntary saccade experiment—Pre- and post-adaptation

Before and after the adaptation, subjects performed one pre- and one post-adaptation block. During these blocks, the display did not jump upon detection of the horizontal voluntary saccade but instead was substituted by the visual mask in the mask condition or by a blank screen in the no-mask condition. Each pre- and post-adaptation block is composed of 12 rightward trials and 12 leftward trials.

### Data analysis

#### Saccade parameters

Horizontal and vertical movements of both eyes were averaged and the resulting “cyclopean eye” signal analyzed off-line with a custom program developed in the Matlab v.7.1 environment (Mathworks, MA., U.S.A.). The position and time of the beginning and end of the horizontal primary saccades were detected on the basis of a velocity threshold of 50°/sec. Additionally, in the voluntary saccade experiment, the termination of the vertical saccade was detected to allow calculation of the latency of the horizontal saccade (i.e. duration of fixation period separating these two saccades). Saccades contaminated by a blink were eliminated from further analysis.

Saccade amplitude was calculated as the difference between the final and initial eye positions, and saccade duration as the difference between the termination and onset times. The gain of horizontal prim°ary saccades was calculated as the ratio between saccade amplitude and retinal error (difference between target position and initial eye position). Mean gain values were calculated for the pre-adaptation block, the 4 adaptation blocks and the post-adaptation block. Saccades with a gain outside the [mean ±3 standard-deviations] range were removed from further analysis. Gain change for the primary saccade of the trial n was calculated as follow:
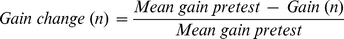



Note that gain changes consistent with the effect of adaptation (i.e. gain decrease) have a positive value.

Secondary saccades produced in the adaptation blocks were also analysed. We measured their latency relative to the end of the primary saccade. Secondary saccades directed to the jumped target will be called corrective secondary saccades (or corrective saccades). In order to see the effect of target duration and visual masking on corrective saccades production, we calculated the rate of occurrence of corrective saccades relative to the total number of secondary saccades.

### Statistical analysis

Statistical analyses were performed with the STATISTICA 9 software package. Initial analyses revealed no effect of saccade direction on the saccade gain in pre-adaptation (t test for reactive and voluntary saccade, p>0.57 and p>0.55, respectively) and on the gain changes during the adaptation (two-ways ANOVAs: no significant interaction between trials block × saccade direction, F[1], [4]<4.6; p>0.10). Thus, the two saccadic directions were pooled for further statistical analyses.

The gain, duration, peak velocity and latency of saccades recorded in pre-adaptation were submitted to two-ways ANOVAs with the “saccade type” (reactive versus voluntary) and “visual masking” (mask versus no-mask conditions) factors.

Then for the mask condition, the mean gain change relative to pre-adaptation was submitted to a three-way ANOVA with the following factors: “block” (pre vs a25 vs b25 vs c40 vs d40 vs post), “saccade type” (reactive vs voluntary) and “target duration” (15 ms vs 50 ms vs 100 ms vs 800 ms). To search for effects of the mask for short durations of jumped target, saccadic gain change was submitted to another four-way ANOVA with the following factors: “block”, “saccade type”, “target duration” (only 15 ms vs 50 ms) and “visual masking” (mask vs no-mask).

The effect of target duration, visual masking and saccade type on visual processing was also assessed by measuring the secondary corrective saccades during the adaptation. In the mask condition, the rate of occurrence of corrective saccades was submitted to two-way ANOVAs with the “saccade type” factor (reactive vs voluntary) and the “target duration” factor (15 ms vs 50 ms vs 100 ms vs 800 ms). For the short target durations (15 and 50 ms), these rates of occurrence were also submitted to three-way ANOVAs with the “saccade type” (reactive vs voluntary), the “target duration” (15 ms vs 50 ms) and the “visual masking” (mask vs no-mask) factors. Significant ANOVAs were followed by post-hoc Fisher's LSD tests. Significance level was set at p<0.05.
